# Enhanced Response of a Proteinase K-Based Conductometric Biosensor Using Nanoparticles

**DOI:** 10.3390/s140713298

**Published:** 2014-07-23

**Authors:** Wided Nouira, Abderrazak Maaref, Abdelhamid Elaissari, Francis Vocanson, Maryam Siadat, Nicole Jaffrezic-Renault

**Affiliations:** 1 Institute of Analytical Sciences, University of Lyon, La Doua Street, 5, 69622 Villeurbanne, France; E-Mail: nicole.jaffrezic@univ-lyon1.fr; 2 Department of Physics, College of Science, Qassim University, Buraidah 51452, Saudi Arabia; 3 Laboratory of Interfaces and Advanced Materials, University of Monastir, Avenue of Environment, 5019 Monastir, Tunisia; E-Mail: abderrazak.maaref@fsm.rnu.tn; 4 University of Lyon, CNRS, UMR 5007, LAGEP-CPE, 43 Bd. 11 Novembre 1918, Villeurbanne, France; E-Mail: elaissari@lagep.univ-lyon1.fr; 5 Hubert Curien Laboratory, University of Lyon, F-42023 Saint-Etienne, France; E-Mail: francis.vocanson@univ-st-etienne.fr; 6 LASC, ISEA, University of Metz, 7 Marconi Street, 57070 Metz, France; E-Mail: maryam.siadat@univ-lorraine.fr

**Keywords:** gold nanoparticles, magnetic nanoparticles, proteinase K, hydrolase, conductometric transducers, response enhancement

## Abstract

Proteinases are involved in a multitude of important physiological processes, such as protein metabolism. For this reason, a conductometric enzyme biosensor based on proteinase K was developed using two types of nanoparticles (gold and magnetic). The enzyme was directly adsorbed on negatively charged nanoparticles and then deposited and cross-linked on a planar interdigitated electrode (IDE). The biosensor was characterized with bovine serum albumin (BSA) as a standard protein. Higher sensitivity was obtained using gold nanoparticles. The linear range for BSA determination was then from 0.5 to 10 mg/L with a maximum response of 154 μs. These results are greater than that found without any nanoparticles (maximum response of 10 μs). The limit of detection (LOD) was 0.3 mg/L. An inter-sensor reproducibility of 3.5% was obtained.

## 1. Introduction

Biosensors can be considered as competitive tools for environmental monitoring because of their specificity, fast response and low cost. The use of nanoparticles (NPs) in biosensors is a relatively new area of research, nevertheless, the literature already shows numerous examples of the incorporation of NPs into biodevices [1–5]. Nanoparticles, specifically gold nanoparticles (AuNPs), are materials of interest in a rapidly developing area of biosensors [6–8]. AuNPs exhibit strong surface plasmon resonance (SPR) that depends on the size of AuNPs and the relative distance between AuNPs [9–11]. We can find nanobiosensors for the specific detection of biologically-relevant molecules like enzymes [12] nucleic acids [13] and proteins [14]. Magnetic nanoparticles (MNPs) have found a lot of applications in immunoanalysis, immobilization, and purification of enzymes, DNA and proteins, and anti-tumor drug transportation and in the conception of biosensors [15–17].

Electrochemical methods of protein detection seem promising with regard to applications in the areas of automatic analysis in complex media due to their simplicity and high sensitivity. Furthermore, unlike optical protein determination methods, the electrochemical sensor is a label-free technique, able to measure turbid and colored samples. In environmental applications, there is great interest in being able to detect proteins *in situ*. In particular, it is important to quantify organic matter contamination of the hyporheic zone that can be assimilated by microorganisms. The three types of organic matter that can be used to monitor the assimilation are the following: lipids (10%–40% of oxidizable organic matter), glucides (10%–15%) and proteins (20%–30%). Among them, the protein content seems to constitute an excellent indicator of the hyporheic activity [18].

Proteinases are involved in a multitude of important physiological processes, such as protein metabolism, blood-clotting cascade and cell apoptosis [19]. Therefore, they can be considered to be biological markers and therapeutic targets for these pathologies. In addition, some proteinases have been used with remarkable success for economically viable, mild and environmentally benign industrial production processes [20]. Consequently, the developments of proteinase assay are of high importance for drug screen, medical diagnostics and the discovery and optimization of industrial enzymes. There are several classes of proteases depending on their active site [21]. For example, trypsin is a serine based protease of only one polypeptide chain of 223 aminoacids. It catalyses the hydrolysis of the peptide connection at the end of the residues of aminoacids, lysine and arginine (basic amino acid). Proteinase K is a serine based protease of 28.9 kDa that hydrolyzes any protein of any origin in a few hours, preferentially the peptide connections located after the hydrophobic amino acids (leucine, for example).

Much work has been done with these conductometric biosensors for the detection of a variety of molecules [22–29]. This type of biosensor presents many advantages: thin-film electrodes are suitable for miniaturization and large-scale production using low cost technology; they do not need any reference electrode and the transducers are not light sensitive. As it has been modeled [30,31], the observed steady-state response of the conductometric biosensor is the result of the reaction rate limited kinetics of the enzymatic reaction and the diffusive flux of enzymatic reaction products away from the transducer surface, in the boundary layer. The most detectable result of enzymatic reaction is pH variation; a pH limit value is reached at the transducer surface.

In this report the authors present the conception of a conductometric proteinase biosensor based on nanoparticles. Protease is immobilized on two types of nanoparticles (AuNPs and MNPs) in order to test the effect on the sensitivity of detection of a model protein, bovine serum albumin (BSA). The relation between type of enzyme and type of nanoparticles for the optimal conception of conductometric biosensor will then be established.

## 2. Experimental Section

### 2.1. Materials

Proteinase K (EC 3.4.21.64, freeze-dried from *Tritirachium album*) with p*I* = 8.9, bovine serum albumin (BSA), glutaraldehyde (GA) (grade II, 25% aqueous solution) were purchased from Sigma–Aldrich (Saint-Quentin-Fallavier, France). All other reagents were of analytical grade and were used without any further treatment. Gold nanoparticles (size 23 nm) were made up and stored in clean glass vials. Chemicals (tetrachloroauric(III) acid trihydrate and sodium citrate dehydrate) used were of highest available purity and were supplied by Aldrich and Sigma. Magnetic nanoparticles, carboxyl-Adembeads type (from Ademtech, Pessac, France) were monodispersed (diameter 200 nm) and these super-paramagnetic nanoparticles were composed of magnetic core (iron oxide content of about 70%).

### 2.2. Sensor Design

The conductometric transducers, consisting of two identical pairs of gold interdigitated thin film electrodes (thickness: 150 nm), were fabricated by vacuum deposition on a ceramic substrate (5 × 30 mm) at the Lashkaryov Institute of Semiconductor Physics (Kiev, Ukraine). A 50 nm-thick intermediate chromium layer was used for better gold adhesion. The dimension of each interdigital space and digit was 20 μm and the length of the digits was about 1.0 mm. The sensitive area of each pair of electrodes was about 1 mm^2^ [32]. Microelectrodes were placed in a glass cell filled with 5 mL of a 5 mM phosphate buffer pH 7.3. The solution was stirred vigorously. Measurements were then performed at 23 ± 2 °C by applying to the differential pairs of electrodes with an alternating voltage (10 mV amplitude, 100 kHz frequency) generated by a low-frequency wave-form generator (SR830 Lock-in amplifier from Stanford Research Systems, Sunnyvale, CA, USA). Measurements based on the detection of solution conductance variations inside membrane. Proteinase K induces catalytic reactions hydrolysing proteins producing different ionic amino-acids resulting in measurable conductivity changes.

### 2.3. Preparation of Protease K Coated Nanoparticles

#### 2.3.1. Preparation and Coating of Gold Nanoparticles

Gold nanoparticles were synthetized according to the following procedure: tetrachloroauric(III) acid trihydrate (11.6 mg, 2.9 × 10^−5^ mol) were dissolved in pure water (21 mL) in a 100 mL round bottomed flask equipped with a condenser. Then this solution was heated to reflux. In another 100 mL round bottomed flask, sodium citrate dihydrate (27.4 mg, 9.3 × 10^−5^ mol) was dissolved in pure water (7 mL) and then added to the first flask. During the addition, the yellow gold chloride solution turned red, indicating the formation of gold clusters. The mixture was moderately stirred, refluxed during 30 min and cooled to room temperature under continuous stirring to yield the nanoparticle solution. Laser granulometer measurements (Malvern Zetasizer 1000 HSA laser granulometer) show the absence of aggregates and a nanoparticle size of around 23 nm with a standard deviation of 5 nm. From the UV-Vis spectrum recorded at room temperature on a Perkin Elmer Lambda 900 UV/Vis/NIR spectrophotometer, the plasmon band is measured at around 527 nm.

A suspension of gold nanoparticles (1% w/w) was firstly centrifuged at 20 °C using a 2–6 K SIGMA centrifuge from Fisher Scientific SAS (Illkirch, France) at 9000 g for 20 min. The supernatant phase was then eliminated and the gold nanoparticles were immersed in 100 μL of aqueous solutions of 5 mg·mL^−1^ of protease (positive charge), under mechanical stirring for 15 min [1]. After the adsorption process, protease-coated gold nanoparticles were removed by similar centrifugation and redispersion in 20 mM of phosphate buffer at pH 7.3.

#### 2.3.2. Preparation and Coating of Magnetic Nanoparticles

1/500 of nanoparticles (suspension 3.03%) was first homogenized under mechanical stirring for 15 min in 100 μL of ultrapure water, twice. The nanoparticles were then coated with protease. This step was performed in 100 μL of aqueous solutions of 5 mg/mL of protease, under similar stirring conditions during 15 min. The nanoparticles were then recovered by applying a mild magnetic field. Protease-coated MNPs were removed under a mild magnetic field and redispersed in 20 mM of phosphate buffer at pH 7.3.

### 2.4. Immobilization of the Enzyme on the Transducer

The enzymatic detection is made possible with the immobilization of the enzyme on the sensor surface by crosslinking of proteinase K coated nanoparticles, with BSA in a saturated glutaraldehyde vapour. The nanoparticles were dispersed in 20 mM phosphate buffer (pH 7.3) containing 6% of BSA, 4% of proteinase K and 10% of glycerol, as optimized in our previous work [24]. After 1 h of mixing, these solutions were centrifuged (for gold nanoparticles) or decanted under magnetic field (for magnetic nanoparticles). After separation and redispersion in 20 mM phosphate buffer, 0.2 μL of these solutions was deposited onto the sensitive area of the working sensor. The reference sensor was functionalized with non-coated negatively charged nanoparticles, dispersed in 20 mM phosphate buffer (pH 7.3) containing 10% of BSA and 10% of glycerol. The sensors were then placed in saturated glutaraldehyde (GA) vapor for 30 min. After exposure, the biosensors were dried at room temperature for15–30 min and stored at 4 °C before the experiments.

### 2.5. Transmission Electron Microscopy Measurements (TEM)

Transmission electron microscopy (TEM) images were obtained with a Phillips CM120 electron microscope (Eindhoven, Netherlands). One drop of highly diluted dispersion was placed onto a copper grid (mesh 200 and covered with formvar-carbon) and dried at room temperature before TEM analysis. The images were registered at 100 kV.

## 3. Results and Discussion

### 3.1. Characterization of Nanoparticles

#### 3.1.1. Gold Nanoparticles

**Figure 1.**
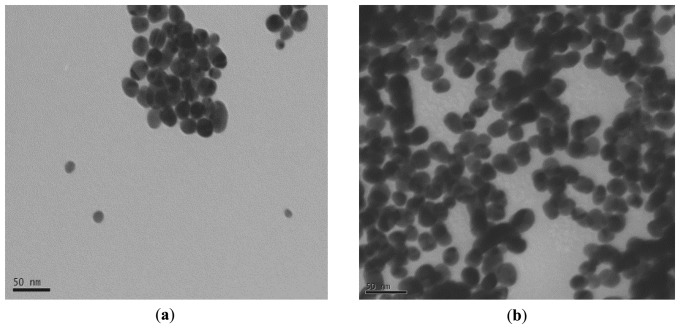
TEM images of gold nanoparticles without (**a**) and with (**b**) proteinase K.

TEM analysis of gold nanoparticles revealed the polydispersity of the particles and also the spherical shape of the majority of the particles. In addition, the seed particles or the gold nanoparticles exhibit the presence of some heterogeneity in chemical composition as clearly observed on TEM images (Figure 1).

In fact, the internal morphology of these gold nanoparticles exhibits the presence of two phases: one organic (transparent) and the second black part can be attributed to the inorganic gold part. The enzyme cannot be clearly observed on the particles probably due to its low electronic density and there is no apparent change in size. Zeta potential of gold nanoparticles was initially negative due to citrate adsorption. After proteinase K coating, it turned to positive (results not shown).

#### 3.1.2. Magnetic Nanoparticles

**Figure 2.**
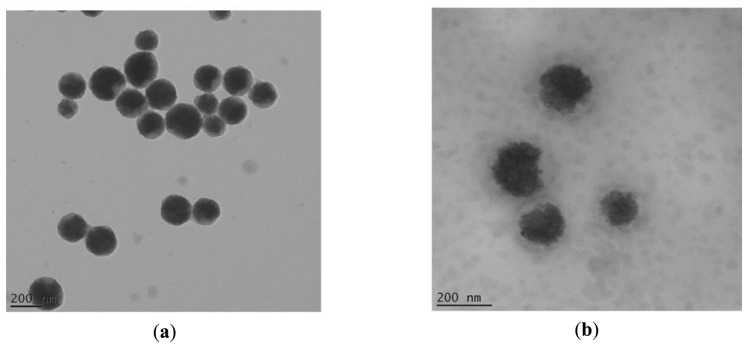
TEM images of magnetic nanoparticles without (**a**) and with (**b**) proteinase K.

The observed TEM images of the manufactured magnetic latex particles revealed first the polydispersity character of the dispersion. The particles are spherical in shape and exhibit almost slight core-shell like morphology with magnetic core and polymer shell (cf. Figure 2). Enzyme cannot be clearly observed and there is no apparent change in size.

Zeta potential of magnetic nanoparticles was initially negative due to carboxylic groups. After proteinase K coating, it turned to positive (results not shown).

### 3.2. Conductometric Biosensor Response

For development of our biosensor, proteinase K coated gold nanoparticles were immobilized on interdigitated conductometric electrodes. Proteases hydrolyse proteins into free amino acids which causes the local conductivity change. An increase of conductivity is observed after every injection of BSA; the response time is τ_90_ = 3 min. After a stable base-line in phosphate buffer solution, injection of BSA stock solutions into blank solution caused significant sensor response due to the enzymatic hydrolyse of the substrate. After all the optimizations, a calibration curve has been established.

**Figure 3.**
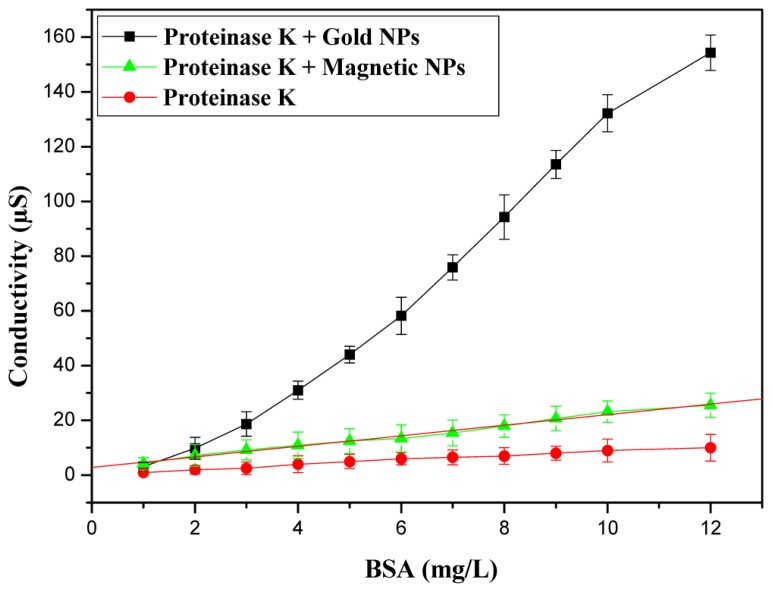
Calibration curves of the proteinase K biosensor, with and without nanoparticles (AuNPs and MNPs).

The calibration curve with AuNPs is presented in Figure 3. The linear range for BSA determination was from 0.5 to 10 mg/L with a maximum response of 154 μs. The limit of detection (LOD) was 0.3 mg/L. In order to investigate the effect of NPs on the amplitude of response of our biosensor, conductometric measurements were performed with protease coated magnetic nanoparticles. Figure 3 shows the calibration curve of the BSA sensor measured under the optimum conditions with MNPs based conductometric biosensor. The response maximum obtained with gold nanoparticles is 154 μs compared to 25 μs for protease coated magnetic nanoparticles. The response maximum obtained with gold nanoparticles is much higher than that found without nanoparticles (maximum response of 10 μs) [23,25].

The conductometric sensor based on proteinase K coated AuNPs was stored at 4 °C. The reproducibility of the biosensor response was tested on five different sensors in PBS solution (5 mM) for the same concentration of proteinase K. The inter-sensor reproducibility is 3.5%.

Concerning the selectivity of this proteinase K based conductometric biosensor, it has already been applied for the detection of model protein [23,25] and even of small peptides [33]; it has been applied for the detection of proteins in natural river waters, a major part of the organic matter [23,25], in good agreement with the classical methods, showing its selectivity for hydrolysis of proteins.

Conductometric transduction was previously used with another hydrolase (urease) [34] and with an oxidase [24]. It was shown that the response of an hydrolase-based biosensor was increased greatly in the presence of gold nanoparticles. These AuNPs, with their large surface to volume ratio, increase the density of immobilized enzyme and behave as nanoelectrodes, decreasing then the probed distance at the vicinity of the transducer surface where the local pH variation is higher. Whereas the response of an oxidase based biosensor was higher increased in the presence of magnetic nanoparticles than with gold nanoparticles. In this case the ionic effect is very much lower and the global effect is more complex.

## 4. Conclusions

In this paper we have shown the feasibility of a biosensor based on interdigitated microelectrodes. The concentration range for detection of the reference protein (bovine serum albumin) with the elaborated protease-coated gold nanoparticles is between 0.5 to 10 mg/L, with a maximum response of 154 μs. The results with magnetic nanoparticles proved that the biosensor response is greater using gold nanoparticles. The effect of high conductivity of AuNPs gives a high amplification in case of hydrolase enzyme.
